# Human molecular evolutionary rate, time dependency and transient polymorphism effects viewed through ancient and modern mitochondrial DNA genomes

**DOI:** 10.1038/s41598-021-84583-1

**Published:** 2021-03-03

**Authors:** Vicente M. Cabrera

**Affiliations:** Retired member of Departamento de Genética, Facultad de Biología, Universidad de La Laguna, Canary Islands, Spain

**Keywords:** Evolution, Genetics

## Abstract

Human evolutionary genetics gives a chronological framework to interpret the human history. It is based on the molecular clock hypothesis that suppose a straightforward relationship between the mutation rate and the substitution rate with independence of other factors as demography dynamics. Analyzing ancient and modern human complete mitochondrial genomes we show here that, along the time, the substitution rate can be significantly slower or faster than the average germline mutation rate confirming a time dependence effect mainly attributable to changes in the effective population size of the human populations, with an exponential growth in recent times. We also detect that transient polymorphisms play a slowdown role in the evolutionary rate deduced from haplogroup intraspecific trees. Finally, we propose the use of the most divergent lineages within haplogroups as a practical approach to correct these molecular clock mismatches.

## Introduction

Evolutionary genetics studies human history within a chronological molecular context. At this respect, its main tool is the molecular clock^1^ which established that the rate of divergence between proteins or DNA sequences, measured as mutational differences between lineages, is proportional to the time elapsed since their initial separation. However, later studies have found that the clock varies with nucleotide or amino acid position within a lineage, also between lineages and, much more, among taxa. Thus, each time more sophisticated models have to be implemented to take into account this heterogeneity^2^. Another problem detected with this molecular timescale is that it varies, intra and interspecifically, along the history of the lineage examined^3,4^. Recently, the progress in the analysis of ancient DNA has provided additional calibrated points at different times to improve the evolutionary rate estimations^5,6^. However, at least in humans, these improvements have not varied substantially the evolutionary rates previously obtained with modern sequences^4,7–10^. Furthermore, the significant differences observed between mutation rate and evolutionary rate estimations have not disappeared when uniparental markers have been substituted by massive whole genome sequencing^11–13^ or when the mutation rate has been substituted by the recombination rate^14^.

Despite of the uncertainty of the evolutionary rate commented above, genetics is playing an authoritative role in the interpretation of human history. Thus, when archaeology places the origin of modern humans more than 300,000 years ago (ya)^15,16^, or its Eurasian expansion from Africa more than 120,000 ya^17–19^, dates significantly older than the genetic estimates (150,000–200,000 and 50,000–60,000 respectively), archaeological findings are considered genetically irrelevant because they may not have contributed to the gene pool of current human populations.

Against that view, we have suggested that accepting a dependency of the divergence rate on past demographic dynamics, and trusting on the average germline mutation rate as a reliable value, it is possible to reconcile the archaeological dates with the genetic ones^20^.

In addition, we will see that the persistence of an ancestral lineage in a population along generations, in spite of the rather fast mtDNA mutation rate, strongly affects the accuracy of the intraspecific coalescence age estimations.

In this study, comparing ancient and modern mitogenome sequences, we confirm that evolutionary rates notably vary along time reaching significantly slower and faster values than the average germline mutation rate. Thus, a time dependency effect exists on the evolutionary rate with increasing acceleration in recent times. We also have detected that transient polymorphisms play a slowdown role in the evolutionary rate estimated from trees. We propose the use of the most divergent lineages within haplogroups as a practical approach to correct these uncertainties.

## Results

### Molecular evolutionary rate

We use 423 ancient mitogenomes (Supplementary Table 1) and 784 modern mitogenomes (Supplementary Table 2 and 3) to estimate the mitochondrial evolutionary rate. The ancient mitogenomes were subdivided into ten periods according to their respective calibrated ages. Mean ages per period ranged from 1119 ± 483 years BP to 40,160 ± 4658 years BP (Table 1). We did not observe significant differences when comparing the mean number of mutation differences between modern and ancient sequences for each macrohaplogroup (M, N, and R) within periods. Unpaired t-test for the most divergent comparison gave a p-value of 0.54. This homogeneity among haplogroups reinforces the statistical confidence of the total values obtained. The observed evolutionary rates range from 4.33 × 10^–8^ (95% CI 3.90–4.82 × 10^–8^) mutations per site per year (msy) for the most recent period to 1.91 × 10^–8^ (95% CI 1.72–2.10 × 10^–8^) msy for the oldest period (40,160 ± 4658 ya). If we equated the former estimation to the last germline mutation rates published 1.30 × 10^–8^ ^21^ or 1.89 × 10^–8^ msy ^22^, our rate is faster by a factor between 2.27 and 3.33-fold respectively. However, the rate obtained from the oldest period of 1.91 × 10^–8^ (95% CI: 1.72–2.10 × 10^–8^), is at the lower range of those calculated by other authors^7–10,23,24^ (Table 2).

**Table 1:** Mutation differences between modern and ancient mitogenomes by macrohaplogroup and period.

Periods	Mean ages BP	Hg M	Hg N	Hg R	Total observed	Expected
First	1119 ± 483	1.67 ± 0.56 (3)	0.34 ± 3.61 (18)	0.65 ± 1.62 (119)	0.63 ± 0.03 (140)	0.63
Second	2652 ± 1025	1.13 ± 1.63 (15)	0.50 ± 1.38 (6)	1.16 ± 2.19 (18)	1.05 ± 0.04 (39)	1.49
Third	4294 ± 372	0.78 ± 1.48 (10)	0.42 ± 1.87 (9)	2.33 ± 2.08 (28)	1.63 ± 0.12 (47)	2.42
Fourth	5164 ± 479	1.52 ± 1.03 (5)	1.35 ± 1.70 (4)	1.51 ± 1.83 (28)	1.51 ± 0.08 (37)	2.91
Fifth	6936 ± 458	1.86 ± 1.79 (8)	2.90 ± 1.93 (8)	3.61 ± 3.01 (32)	3.20 ± 0.09 (48)	3.9
Sixth	8504 ± 822	3.00 ± 2.83 (2)	3.08 ± 2.39 (9)	3.36 ± 2.02 (29)	3.28 ± 0.02 (40)	4.79
Seventh	13,017 ± 1533	4.25 ± 0.97 (5)	2.60 ± 0.14 (2)	4.59 ± 2.31(17)	4.35 ± 0.12 (24)	7.33
Eighth	15,977 ± 1254	NA	NA	6.50 ± 1.50 (11)	6.50 ± 1.50 (11)	8.99
Nineth	29,638 ± 3020	10.00 ± 0.71 (2)	NA	11.23 ± 2.28 (13)	11.07 ± 0.01 (15)	16.69
Tenth	40,160 ± 4658	14.13 ± 2.31 (3)	13.00 ± 3.61 (5)	15.67 ± 4.97 (6)	14.39 ± 0.33 (14)	22.61

() pairs compared.

**Table 2:** MtDNA evolutionary rate estimations.

Evolution rate × 10–8	95% CI/HPD × 10–8	References
1.67	1.37–1.97	^23^
1.91	1.72–2.10	This study
1.92	1.16–2.68	^8^
2.23	1.71–2.75	^7^
2.4	1.70–3.20	^10^
2.53	1.80–3.20	^24^
2.67	2.16–3.16	^8^
2.74	2.44–3.01	^9^
4.33	3.90–4.82	This study

### Time dependency effect

**Figure 1:**
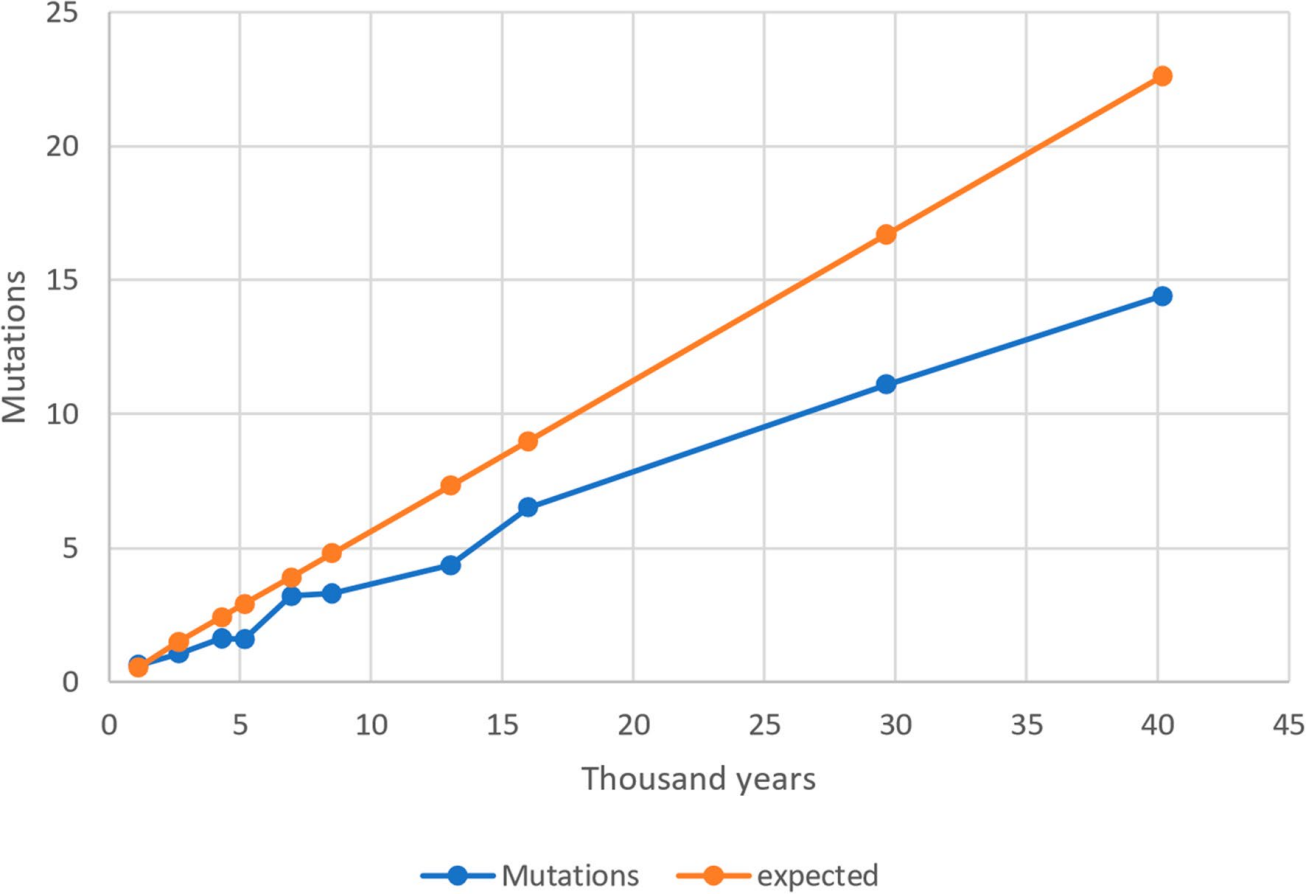
Observed and expected mean number of mutations accumulated along periods on the y-axis and time measured in thousand years on the x-axis.

Assuming a linear accumulation of mutations through time, we should expect the same mutation rate for both the youngest and the oldest periods but, in reality, the observed value for the oldest is 2.27-fold slower than that calculated for the youngest. This is compatible with the existence of a time dependence effect on the rate estimates with significantly faster substitution rates in recent times compared to the older ones as observed by other authors in humans and other species^3,4,7,25^. This effect is graphically represented in Fig. 1 where the observed and expected number of mutations accumulated along periods are plotted against time measured in thousand years. In fact, we observed a significantly negative regression of the age of each period against the evolutionary rate observed at the same period (R = − 684.8; p = 0.0001).

### Transient polymorphism effect

**Figure 2:**
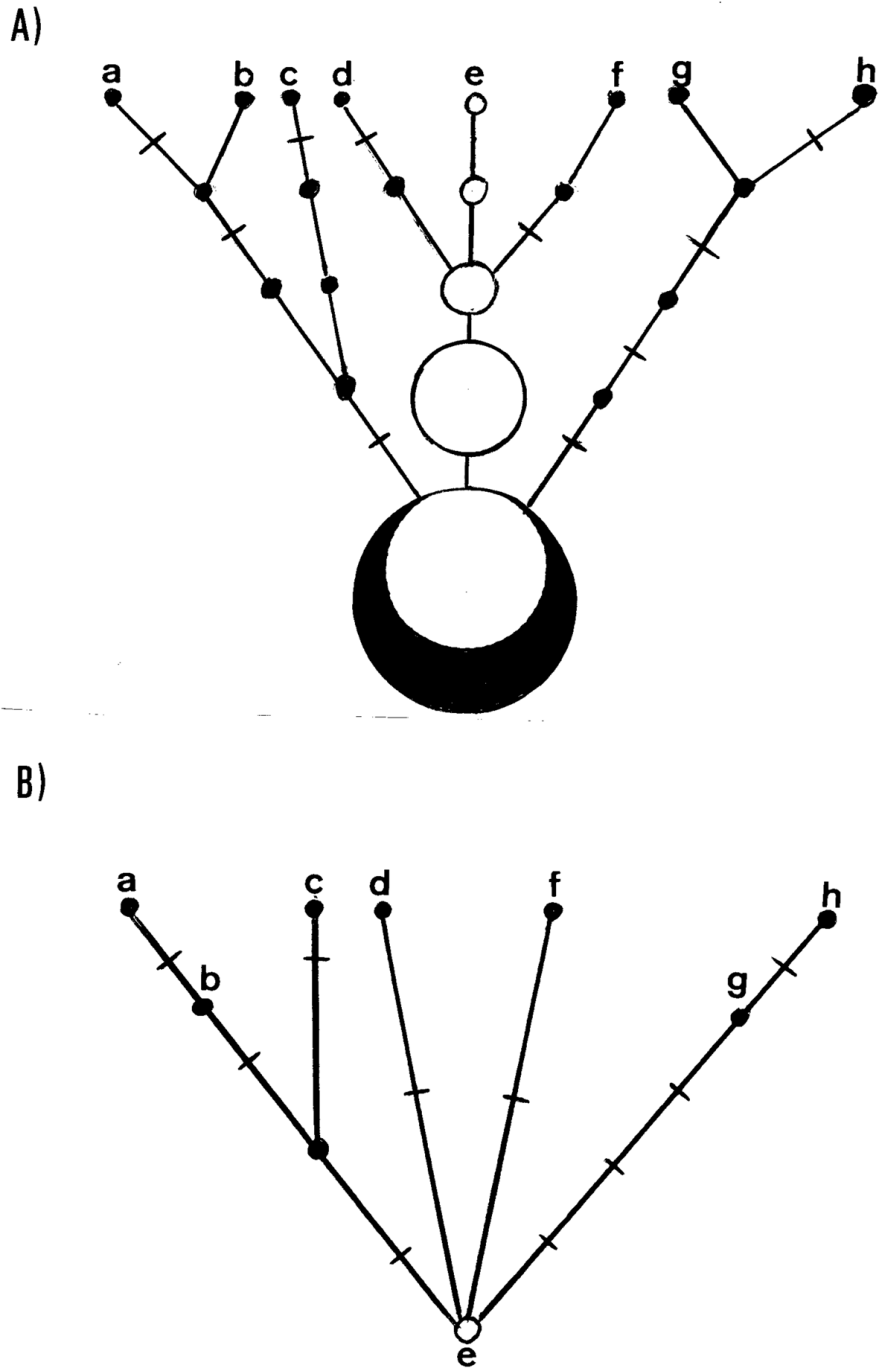
Comparison between a genealogy (**A**) and a tree (**B**) constructed from the same sample (a to h). White circles are individuals with the ancestral lineage, black circles are individuals with additional mutations. Inter-circle segments represent generations and crosses on the segments represent mutations.

We have seen in “Methods” that the persistence of a transient mtDNA polymorphism approximates to 2Ne, thus, supposing an effective size of 5000 females in humans, the process would last about 10,000 generations or, giving a mean generation time of 25 years, around 2.5 × 10^5^ years. An immediate consequence is that in a growing population such as the human^26^, the fixation time and, therefore, the persistence of a transient polymorphism would be even longer^27^. Therefore, it is expected that, in spite of the usually high mitogenome diversity found in human populations, there can be large sets of individuals sharing the same mitogenome in them^28^. For example, an ancient mtDNA study has corroborated empirically the persistence of an ancestral M lineage unaltered along a period of more than 8000 years^29^. In addition, Using an online Poisson Distribution Calculator (https://www.stattrek.com ) and an average rate of mutation success of 1.947 × 10^–4^ per genome per year, the probability that an ancestral mitogenome does not suffer any mutation in a period of 12,500 years (P(x) = 0) is still non-significant (P = 0.08771), which is very similar to the limit of 500 meiosis/generations (12,500 years assuming 25 years per generation) found as the time two individuals sharing the same mitogenome in a population are matrilineally related^28^. This period may increase in cases where a population suffers a major bottleneck or when a new population is founded from a few individuals and only a single lineage or a majority lineage remains. Consequently, a lineage could remain immutable for several generations while identical lineages in the same population suffer one or several mutational changes in the same time interval. We represent such a scenario in Fig. 2A as a maternal genealogy. Notice that the ancestral lineage can give rise to offspring in different generations throughout its existence in the population. In this way, when the population is sampled after n generations, we can find, in addition to the ancestral lineage (e), lineages derived from it that have accumulated significant mutational differences in their branches (f, h). However, this fact is not reflected in the tree built from the same sample (Fig. 2B) because, irrespective of the generation in which they appeared, all derived lineages sprout at the same time from the ancestral node (e). This difference between genealogies and trees has notable consequences. On the one hand, it could explain the lack of mutation rate homogeneity between lineages found in intraspecific haplogroup comparisons^30–33^ without the necessity to invoke selective constrains^34^. On the other hand, the lack of representation of the generations passed without mutational impact on the lineages from which the trees are built, has the general effect of shortening the ages of coalescence calculated from these trees.

One simple way to attenuate this shortening effect is to choose those sequences with the major number of mutations from the haplogroups analyzed instead of the mean of their sequences, or, taken into account the variance of the Poisson distribution, taking the mean of the sequences grouped in the lower interquartile. In supplementary Table 3 we have grouped those sequences with the highest number of mutations for the main haplogroups constituting the African L0 and L3 macrohaplogroups and the out-of-Africa macro-haplogroups M, N and R. With these data and using the evolutionary rates mentioned in “Methods” we calculated coalescence ages for important episodes of the human history, as the age of modern humans (L0–L1′2′3′4′5′6′7 split), the out of Africa (L3′4 split), the return to Africa of Eurasian populations (L3 coalescence), the expansion of modern humans throughout Eurasia (M, N, and R coalescences), with special emphasis in the colonization of the Sahul (P coalescence), Europe (U5 coalescence), and the Americas (A2, B2, C1, D1, D4h3a, and X2a coalescences). As expected, all these new estimations are significantly older than those calculated previously (Table 3).

**Table 3:** Coalescence ages in thousand years for some major events in human history (95%CI/HPD).

	Soares et al.^23^	Fu et al.^8^	Rieux et al.^7^	Cabrera^20^	This study Soares/amtDNA
MtEva	192 (152–234)	157 (120–197)	143 (112–180)	318 (283–353)	312 (308–316)/211 (208–214)
L3′4	86 (66–106)	na	na	166 (140–191)	150 (142–158)/102 (96–108)
L3	73 (51–95)	78 (62–95)	72 (54–93)	112 (133–92)	122 (102–143)/83 (69–97)
M	55 (43–67)	77 (61–93) a	59 (49–70) b	na	116 (107–124)/79 (73–85)
N	64 (50–78)	77 (61–93) a	58 (51–66) b	na	119 (115–122)/80 (78–83)
R	60 (47–74)	na	na	na	128 (124–131)/86 (84–89)
P	na	54 (43–66)	57 (43–71)	112 (91–133)	101 (87–115)/69 (59–78)
U5	36 (25–47)	30 (22–37)	50 (39–62)	na	63 (57–70)/43 (39–47)
A2	13 (9–18)	na	30 (22–40)	na	41 (37–44)/28 (25–30)
B2	15 (9–20)	na	23 (16–31)	38 (25–50)	46 (37–55)/31 (25–37)
C1	17 (12–26)	na	31 (22–41)	na	30 (24–37)/21 (16–25)
D1	14 (9–18)	na	31 (22–40)	na	32 (17–47)/22 (12–32)
D4h3a	na	na	na	na	26 (22–29)/17 (15–21)
X2a	13 (6–20)	na	21 (14–30)	na	22 (14–31)/15 (10–21)

## Discussion

While the mtDNA mutation rate, despite the environmental and genetic variables to which it is subject^35^, approximates to a consensual average value^21,22^, we have verified here that the substitution rate of mtDNA varies over time and can be less than or greater than the mutation rate. Thus, we have reached to the same situation as for the whole genome for which it was found that the evolutionary rate doubles the mutation rate^11^ questioning the chronology of human evolution^36,37^. However, we should expect this to be the case if we admit that the evolutionary rate is dependent on the population size^20^ and that there have been enormous differences in the human population size throughout its demographic history with explosive population growth in recent times^26^. This apparent contradiction with the neutral theory of molecular evolution deserves some clarification. The first is to differentiate between the rate of transmission and the fixation rate.

When a mutation appears in a population of size N_0_ it has a probability of being transmitted to the next generation (N_1_) of 1/N_0_, and a probability of fixation, given that it is transmitted, of 1/N_1_. If the population size remains constant along generations (N_0_ = N_1_) the probability of transmission and the probability of fixation is the same just as the neutral theory predicts^38^. However, when the population size fluctuates across generations the probability of transmission behaves just the opposite as the probability of fixation. Suppose the case that the population size doubles in the next generation (N_1_ = 2N_0_), then the probability of transmission is 2/N_0_ (it has two chances of being transmitted). However, the probability of fixation of the mutation, given that it is transmitted, is 1/2N_0_. On the contrary, if the population decreases by half in the next generation (N_1_ = N_0_/2) the probability of transmission is 1/2N_0_ but the probability of fixation, given that it is transmitted, is 2/N_0_. Again, this is according to the neutral theory that predicts that in a large population the probability of fixation of a mutation is lower than in a small population. However, it is the rate of transmission which drives the evolutionary rate.

When we estimate the evolutionary rate between populations or species, we are counting mutation differences between sequences regardless of whether these variants are fixed for different alleles or segregate as transient polymorphisms in each population/species. So, what really matters is the amount of variation accumulated independently in each lineage. Indeed, mutations appear according to the mutation rate, but it is known that in a growing population there are more lineages, more mutations accumulate per lineage and these lineages are maintained for longer time than in a decreasing population^27,39^. This explains why in a growing population the evolutionary rate accelerates and can be higher than the mutation rate, since the mutations that appear are more likely to be transmitted and those that already segregate in the population remain polymorphic for a longer time. Just the opposite occurs in a decreasing population.

The time dependency effect, confirmed here by the comparative analysis of ancient and modern mitogenomes, empirically corroborates the arguments exposed above about the dependency of the divergence rate on the population size dynamics. It is indubitable that selection also plays an important role in the rate of evolution, as repeatedly demonstrated by the differences in the evolutionary rate of synonymous versus non synonymous substitutions^40–42^. However, its effect may be explained into the nearly neutral theory of molecular evolution that gives to genetic drift the main role on molecular evolution^43^.

Finally, although we proposed elsewhere a new algorithm to counterbalance the effects of the exponential growth observed in the human population^20^, it is based on the topology of the tree grouping the sampled lineages. However, we have showed here that trees ignore the persistence of ancestral lineages along time with the result of giving coalescent ages younger than the real ones. Although we have proposed to take the lineages with the major number of mutations within each haplogroup instead of the average, as a practical approach to estimate the real coalescence times, more sophisticated models should be implemented to cope with the problems that fluctuating population sizes and tree constrains impose to the evolutionary rate.

Nevertheless, it deserves mention that the two approaches proposed by us have given coalescence times for the origin of modern humans around 300,000 years ago (Table 3) which is more into line with recent archaeological findings^15,16^. With a similar delay, the out-of-Africa of modern humans has been estimated as early as 150,000 years ago a time that could match the archaeological records of Skull and Hafez or even those of the Misliya cave in Israel^18,19^. Also the coalescence of the Eurasian macro-haplogroups M, N and R would delay the human occupation of Eurasia as early as around 100,000 years ago which coincides with the estimated age for human dental remains excavated in China^44^. The split of the Australasian haplogroup P seems to indicate that modern humans reached Sahul at the same time that east Asia (Table 3). As part of this coeval expansion, Eurasian populations might have also returned to Africa coinciding with the African L3 split (Table 3)^45^. Finally, although we proposed a unique migration for the colonization of the Americas around 40,000 years ago^20^ which is directly or indirectly supported by archaeological dates^46,47^, it seems possible that this first migration, signaled by the ages of haplogroups A2 and B2 was followed afterwards by a second wave, also before the Last Glacial Maximum, marked by haplogroups C1, D1, D4h3a and X2a around 27,500 years ago (Table 3).

The evolutionary rate in humans persists as an open question. However, we think there is enough evidence to question the ages proposed by geneticists for the main events in human evolution.

## Methods

### Materials

Publicly available ancient and modern complete human mtDNA sequences were downloaded from the following databases: NCBI GenBank (www.ncbi.nlm.nih.gov/genbank/), Mitomap (www.mitomap.org/MITOMAP), IanLogan 2020 (www.ianlogan.co.uk/sequences_by_group/haplogroup_select.htm), and AmtDB (http://amtdb.org) ^48^.

### Methods

MtDNA haplotypes extracted from the literature were transformed into sequences using the Haplosearch program (http://www.haplosite.com/haplosearch/process/) ^49^. Sequences were manually aligned and compared to the revised Cambridge Reference Sequence (rCRS)^50^ using the BioEdit Sequence Alignment program^51^. Sequence assignment to haplogroups were confirmed using PhyloTree build17 version (http://www.phylotree.org) ^52^. Indels around nucleotides 309, 522, and 16,193 and the hotspot 16,519 mutation were excluded from all analyses. Sequences with the maximum number of mutations for each haplogroup were chosen, after the alignment analysis, within their maximum range excluding clear outsiders. Ancient sequences could be grouped into ten different periods with sufficient statistical sample size. The mean age of each ancient period was defined as the weighted average of the calibrated ages of their sequences. For each time period grouping ancient sequences, we constructed an equivalent modern group with sequences phylogenetically equivalent to those present in the ancient group. The age assigned to all modern sequences was 2000 years AD. To compare our coalescence ages with those of other authors we used the rho statistic^53^ and two substitution rates; the one proposed by Soares et al.^23^ based on modern mitogenomes, corrects for purifying selection and assumes a rate of one mutation every 3624 years, the other is a mean rate of those calculated by several authors using ancient mitogenomes^7–10,24^, resulting in one mutation every 2454 years.

### Molecular evolutionary rate

Molecular evolutionary rates (θ) were calculated using the frequentist estimator:

$$ \theta  = \frac{{\sum _{1}^{{\text{n}}} {\text{dij}}}}{{{\text{nT}}}} $$

where d_ij_ is the number of differences between pairs of modern an ancient sequence, n, the number total of pairs compared and, T, the time of the period analyzed in years. We reasoned that since mutations occur at sequences following a Poisson distribution, within the same lineage, modern sequences should accumulate more mutations than ancient ones as the molecular clock predicts^1^. Even at intraspecific level, most sequences suffer recurrent and parallel mutations mainly at highly variable positions that could misinterpret the real number of mutation differences between sequences. Fortunately, in most of the cases the changes of state of these positions can be hierarchically mapped from the basal branches to the tips of the highly contrasted human mitochondrial DNA phylogenetic tree^52^. Evolutionary rates for each time period were calculated for each macrohaplogroup (M, N, and R) and for the total, estimated as the weighted average of those obtained for each macrohaplogroup.

### Time dependency effect

The time dependency effect on the evolutionary rate was estimated by regression between the mean age of each period and its corresponding evolutionary rate.

### Persistence of a transient polymorphism

Under the neutral theory conditions, the average number of generations between the occurrence and fixation of a mutant, as long as it is not fixed during the process, was calculated as 4Ne^54^. The value is reduced to 2Ne for a haploid system as the mtDNA. This is also the mean time of persistence of a transient polymorphism. On the other hand, new mutations will appear on individual sequences at a mutation rate µ during a dT temporal interval following a Poisson distribution.

The most recent estimations of the human germline mtDNA mutation rate are 1.30 × 10^–8^ ^21^ or 1.89 × 10^–8^ ^22^ mutations per site per year (assuming a generation time of 25 years). Consequently, we are using here an average rate of mutation success of 1.947 × 10^–4^ per genome per year.

## Supplementary Information

Supplementary Information
